# Taking a HIF pill for old age diseases?

**DOI:** 10.18632/aging.101395

**Published:** 2018-03-01

**Authors:** Stefan Kaluz, Chalet Tan, Erwin G. Van Meir

**Affiliations:** 1 Department of Neurosurgery, Emory University, Atlanta, GA 30322, USA; 2 Department of Hematology and Medical Oncology, Emory University, Atlanta, GA 30322, USA; 3 Winship Cancer Institute, Emory University, Atlanta, GA 30322, USA; 4 Department of Pharmaceutics and Drug Delivery, University of Mississippi, Oxford, MS 38677, USA

**Keywords:** hypoxia, hypoxia-inducible factor, atherosclerosis and cardiovascular disease, osteoporosis, arthritis, retinal pathologies, cancer

Aging organisms gradually accumulate molecular and cellular changes that result in a progressive, generalized impairment of organ functions. In humans by age 60, non-communicable diseases that include cardiovascular diseases, chronic respiratory disorders, cancer, and dementia along with age-related losses in hearing, seeing and moving are the major causes of disability and death. Improved understanding of the underlying molecular processes may suggest interventions for preventing, delaying, alleviating or even reversing aging-associated diseases and health decline [1].

Cells in metazoan species produce energy via oxidative phosphorylation, a process that requires a carbon source and oxygen (O_2_). O_2_ homeostasis is therefore of utmost importance and is maintained by intricate circulatory and respiratory systems. When the function of these is compromised, cells in the afflicted areas experience lower than optimal physiological O_2_ levels, a condition termed hypoxia. To cope with hypoxia, cells employ an evolutionarily conserved pathway controlled by hypoxia-inducible factors (HIFs). HIFs are heterodimeric transcription factors formed by an O_2_-regulated α subunit paired with a constitutively expressed HIF-1β subunit. In the presence of O_2,_ the stability and activity of HIF-α subunits are negatively regulated at the protein level by two different O_2_-dependent hydroxylation events. Hydroxylation of specific prolines tags them for ubiquitination and proteasome-dependent degradation, while hydroxylation of a C-terminal asparagine blocks their binding to CBP and p300 co-factors, two histone acetyl transferases that enable transcription by relaxing the chromatin and recruiting RNA polymerase II complex. Under hypoxia, HIF-α subunits escape negative hydroxylation, become stabilized, translocate and dimerize with HIF-1β in nucleus, bind to hypoxia-responsive elements on target genes, recruit coactivators p300/CBP, and initiate transcription. Proteins encoded by hypoxia-inducible genes are functionally diverse, their primary role is to reprogram the cell towards survival under a hypoxic microenvironment and trigger specific physiological responses to help organisms adapt to conditions such as high altitude by inducing synthesis of erythropoietin, a hormone that stimulates production of red blood cells, or wound healing by activating secretion of angiogenesis-stimulating factors such as VEGF [2].

**Figure 1:**
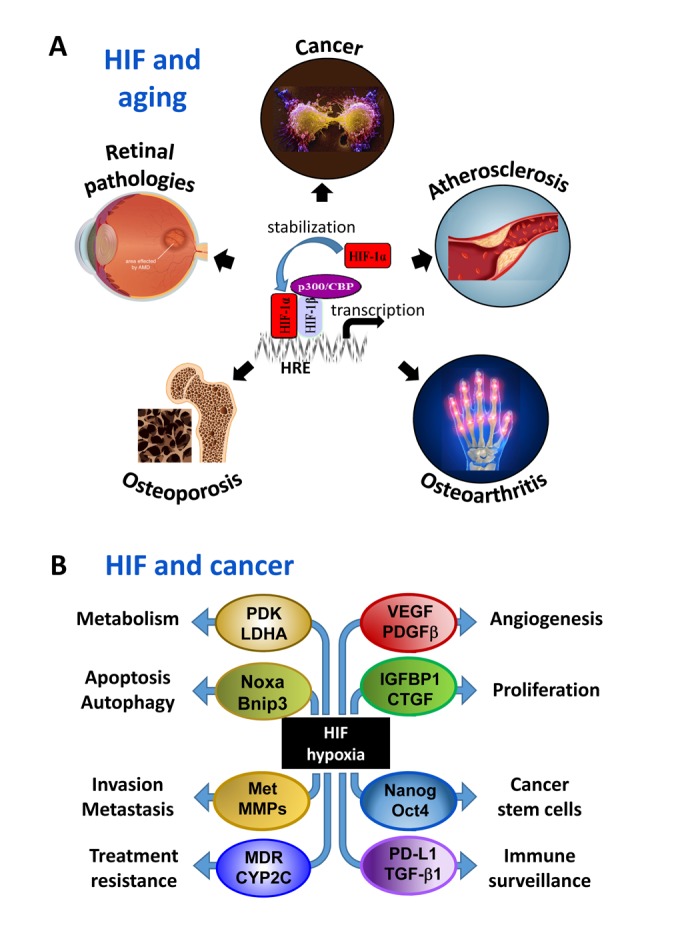
(**A**) Involvement of hypoxia and HIFs in aging-associated diseases; (**B**) Involvement of HIFs in hallmarks of solid tumors.

This fine-tuned physiological response to hypoxia can, however, also be co-opted and contribute to age-related diseases (Figure1A).

Cancer. Hypoxia and HIFs play a critical role in fundamentally important features (hallmarks) of solid tumors, such as increased cell proliferation, survival, and angiogenesis, decreased immunosurveillance, metabolic reprogramming, tumor invasion and metastasis, treatment resistance, and maintenance of cancer stem cells through HIF-mediated upregulation of a plethora of factors (Figure 1B). Activation of the HIF pathway correlates with poor prognosis in cancer patients and HIF is therefore considered a therapeutic target in various types of cancer [2].

Atherosclerosis and cardiovascular disease. Atherosclerotic plaques form in the inner layer of an artery wall as a result of deposition of degenerative material consisting of macrophage cells, lipids, calcium, and fibrous connective tissue. Hypoxia and HIF-1 may participate in the pathogenesis of atherosclerosis by leading to the production of factors that elevate synthesis (3-hydroxy-3-methylglutaryl-CoA reductase, Lipin1) and uptake (CD36, fatty‐acid‐binding proteins, LDLR, VLDLR) of lipids, recruit inflammatory cells (chemokine monocyte chemoattractant protein-1 MCP-1/CCL2), and promoting angiogenesis (VEGF) within plaques. Depending on affected arteries, severe atherosclerosis can inflict coronary artery disease, stroke, peripheral artery disease, or kidney problems [3].

Osteoporosis. Post-menopausal osteoporosis promotes bone loss and predisposes to bone fragility. In pre-menopausal conditions, estrogen protects bones and decreases HIF-1α protein levels in osteoclasts through an estrogen receptor α-dependent HIF-1α destabilization mechanism. Under menopausal conditions, estrogen deficiency allows HIF-1α protein stabilization in osteoclasts, induction of the marker Ctsk that leads to osteoclast activation and bone loss. Systemic administration of a HIF-1 inhibitor as well as osteoclast-specific conditional HIF-1α inactivation protects mice from estrogen deficiency-induced bone loss. Therefore, HIF-1 represents a potential therapeutic target to prevent osteoclast activation and bone loss in post-menopausal patients [4].

Osteoarthritis. In osteoarthritis (OA), articular cartilage is progressively destroyed and other structures of the joints are affected. Prostaglandin E_2_, synthesized by mPGES-1, induces matrix-degrading enzymes and thereby stimulates catabolism of cartilage. HIF-1 activates *mPGES-1* gene expression in chondrocytes and contributes to the excessive catabolism underlying cartilage destruction and OA [5].

Retinal (ocular) pathologies. In addition to its role in eye cancers, HIF has been implicated in a number of ophthalmologic pathologies (e.g. age-related macular degeneration (AMD), diabetic retinopathy, and retinal ischemia), specifically through promoting angiogenesis. AMD is a condition where the macula, the part of the retina that processes the center of the visual field, is progressively damaged. AMD is the leading cause of blindness in the developed world, and ischemia and upregulated VEGF expression are involved in the neurovascular (“wet”) form of late AMD where abnormal blood vessel growth causes vision loss [6]. Anti-VEGF antibody is used for treating ocular neovascularization with less than 50% response rate. Data from animal models suggest that other angiogenic factors activated by HIF (e,g. SDF1, PGF, PDGFB, and SCF) might also contribute to the pathophysiology of AMD; hence, targeting HIF rather than just VEGF is expected to increase therapeutic efficacy [2].

Targeting the HIF pathway in aging. Overactivation of the HIF pathway in cancer raised significant interest in its targeting with small-molecule inhibitors. Most HIF inhibitors affect HIF-α indirectly, preventing HIF-α upregulation through a block in translation, have pleiotropic effects, or are toxic when systemically administered. Novel types of more specific inhibitors with defined mechanism of action are required [7,8]. A few recent compounds inhibit the interaction between HIF-α and HIF-1β or with coactivators p300/CBP, and the most recent one can directly bind HIF-2α. In the context of aging-associated diseases, more work is required to establish whether activation of HIF plays a causative role or is the consequence of some other underlying changes. In either case, there is evidence that targeting HIF-1, rather than its targets, e.g. VEGF, in at least some of these conditions may provide broader effect and eventually translate into greater therapeutic efficacy [2]. As the number of age-associated maladies with activated HIF increases, it is rational to consider whether combining early detection with a HIF inhibitory pill could be of benefit for preventive treatment.

However, the relationship between HIF and aging is more complex. Genetic studies mainly in invertebrates have shown that HIF might control normal physiological processes that both promote and limit longevity. Life span extension imparted by stabilized HIF-1 occurs by a mechanism genetically distinct from both insulin-like signaling and dietary restriction (Leiser et al. 2010). On the other hand, increased life span of *hif-1* deletion mutants was explained in terms of either activation of stress-regulated transcription factor DAF-16 or reactivating endoplasmic stress resistance downstream of mTOR (Leiser et al. 2010). Further studies are warranted to understand the role of HIF-1 in longevity in mammals before merit of therapeutic modulation of its activity for age-related disease can be assessed.
